# Pharmaceutical Compounding in Veterinary Medicine: Suspension of Itraconazole

**DOI:** 10.3390/pharmaceutics16050576

**Published:** 2024-04-24

**Authors:** Gema J. Cabañero-Resta, Bárbara Sánchez-Dengra, Alejandro Ruiz-Picazo, Marival Bermejo, Virginia Merino, Isabel Gonzalez-Alvarez, Marta Gonzalez-Alvarez

**Affiliations:** 1 Departamento de Farmacia y Tecnología Farmacéutica y Parasitología, Universitat de València, Vicente Andrés Estelles s/n, Burjassot, 46100 Valencia, Spain; gema.cabanero.resta@gmail.com (G.J.C.-R.); virginia.merino@uv.es (V.M.); 2 Instituto Interuniversitario de Investigación de Reconocimiento Molecular y Desarrollo Tecnológico (IDM), Universitat Politècnica de València, 46100 Valencia, Spain; 3 Engineering: Pharmacokinetics and Pharmaceutical Technology Area, Miguel Hernandez University, 03550 San Juan de Alicante, Spain; barbara.sanchezd@umh.es (B.S.-D.); alejandro.ruizp@umh.es (A.R.-P.); mbermejo@umh.es (M.B.); marta.gonzalez@umh.es (M.G.-A.)

**Keywords:** compounding, veterinary, itraconazole, excipients, in vitro dissolution, permeability

## Abstract

Itraconazole is a drug used in veterinary medicine for the treatment of different varieties of dermatophytosis at doses between 3–5 mg/kg/day in cats. Nevertheless, in Spain, it is only available in the market as a 52 mL suspension at 10 mg/mL. The lack of alternative formulations, which provide sufficient formulation to cover the treatment of large animals or allow the treatment of a group of them, can be overcome with compounding. For this purpose, it has to be considered that itraconazole is a weak base, class II compound, according to the Biopharmaceutics Classification System, that can precipitate when reaching the duodenum. The aim of this work is to develop alternative oral formulations of itraconazole for the treatment of dermatophytosis. Several oral compounds of itraconazole were prepared and compared, in terms of dissolution rate, permeability, and stability, in order to provide alternatives to the medicine commercialized. The most promising formulation contained hydroxypropyl methylcellulose and β-cyclodextrin. This combination of excipients was capable of dissolving the same concentration as the reference product and delaying the precipitation of itraconazole upon leaving the stomach. Moreover, the intestinal permeability of itraconazole was increased more than two-fold.

## 1. Introduction

Itraconazole is an azolic drug that can be used in veterinary medicine for the treatment of superficial or systemic mycosis [1], i.e., various dermatophytosis generated by fungi of the Malasezzia family [2,3], blastomycosis and histoplasmosis in dogs, or even in aspergillosis or coccidiomycosis in penguins and sea lions, respectively [3,4,5,6]. The drug acts by altering the fungal membrane, inhibiting the synthesis of ergosterol, resulting in the increased permeability of the membrane that causes a loss of cellular content [7,8]. Dermatophytosis is spread by direct contact with the fur or the skin of infected animals, so it can affect a great number of animals in feline colonies or kennels [1,2,3].

Itraconazole is a lipophilic drug capable of accumulating in skin and hair, where it can reach concentrations ten times higher than plasma levels [9]. Itraconazole is metabolized in the liver, and it is eliminated in the bile. It is administered in various periods that are combined with rest periods; both the dose and the rest period in between are dependent upon the species. For cats, the dose ranges from 3 to 5 mg/kg/24 h for twenty-one days (administered in three periods of a 7-day treatment repose) [2,3,10].

In Spain, for the treatment of dermatophytosis caused by different species of fungi, the only authorized medication for veterinarian use is Itrafungol^®^ [11]. This is an oral suspension that contains 10 mg/mL of itraconazole, only authorized for its use in cats, and the only presentation available is 52 mL [9,10].

Due to the fact that Itrafungol^®^ has only one commercial presentation available on the market, compounding would solve some problems associated with that fact. In some situations, the dose of that commercial presentation is not enough to complete an individual treatment, due to the size of the animal or the length of the treatment. The inconvenience is increased if a great number of animals have to be treated. Compounding would permit the adaptation of the concentration and volume to the population to be medicated [2,3,10]. In this sense, formulations of various concentrations as 10 mg/mL, 20 mg/mL, or 40 mg/mL are collected in compounding references [12,13,14,15,16,17].

In veterinary practice, it is preferable to use oral liquid forms or chewable formulations to administer medicaments because animals tend to have swallowing problems or refuse solid medicaments (capsules or tablets). In addition, liquid oral forms allow better adjustment of dose or adaptation of organoleptic characteristics to the target species, aspects that can increase treatment adherence [15,16].

In the case of itraconazole, the most suitable and easiest pharmaceutical form for administration is the oral suspension. It is recommended to administer this drug on an empty stomach, since food can interfere with its absorption [17]. According to the Biopharmaceutical Classification System, itraconazole belongs to class II, which presents serious difficulties when developing new formulations, due to its low solubility [9,18].

Oral suspensions are aqueous-based, multi-dose liquid pharmaceutical forms that have viscous consistency and flavoring agents, such as sweeteners. When selecting excipients with these functions in veterinary practice, it is important to take into account the target species to avoid toxicity problems and be able to reach the desired effect [15,19,20,21,22,23]. Moreover, as itraconazole is a weak base, it needs an acidic medium that facilitates its solubilization and consequent absorption [9].

The objective of the present study was to prepare various compounded formulations of itraconazole at 10 mg/mL. This dose was selected for the present study to compare the formulas with the reference medicine (Itrafungol^®^) in terms of physical stability and absorption, aiming to come up with alternatives to the commercialized formulation that can be produced on a small scale if necessary.

## 2. Materials and Methods

### 2.1. Drugs and Products

The reference product (Itrafungol^®^ oral solution) was acquired from a local pharmacy. Itraconazole and Syrspend pH 4 Dry were purchased from Fagron Ibérica. Polyethylene glycol 400, hydrochloric acid 37%, hydroxypropyl methylcellulose (HPMC), and sodium benzoate were obtained from Sigma Aldrich (Barcelona, Spain). Polysorbate 80, sorbitol liquid 70%, propylene glycol, and sodium carmellose 1500–4500 were obtained from Guinama S.L.U. (Valencia, Spain). Sodium sulfonyl ether β-cyclodextrin was obtained from Fismer Scientific, and acetonitrile (ACN), methanol, and trifluoroacetic acid (TFA) were obtained from Panreac^®^ (Barcelona, Spain).

The amber bottles of 60 mL for the packaging were provided by Guinama S.L.U.

### 2.2. Preparation and Characterization of Itraconazole Formulations

Six different oral suspensions (A to F), whose compositions are shown in Table 1, were prepared and compared with the reference product.

Formulations were prepared according to the following protocols:

- Formulation A: Weigh the required amount of itraconazole and combine it with propylene glycol. Add the required amount of sorbitol and mix. Add the required amount of water and stir until well mixed. Disperse the sodium carmellose by adding it to the dissolution little by little under slow stirring, and keep it under agitation for another 30 min. Let it hydrate for 24 h and mix slowly again for 10 min until the gel is obtained. A viscous white suspension was obtained [10,22].
- Formulation B: Weigh the required amount of itraconazole and add it to the Syrspend pH4 Dry pot. Fill it to 200 mL with purified water and mix well. A viscous white suspension was obtained [17].
- Formulation C: Weigh the required amount of itraconazole and combine it with propylene glycol. Adjust the pH of the mixture with hydrochloric acid to pH 2.5. In another beaker, weigh the sulfonyl β-cyclodextrin, add half of the amount of purified water, and stir until well mixed. Add the cyclodextrin dispersion to the itraconazole mixture under constant stirring. Add purified water until the final volume and continue mixing. A white suspension was obtained [18].
- Formulation D: Weigh the required amount of itraconazole and combine it with polysorbate 80. Added the polyethylene glycol 400 and adjust the pH of the mixture with hydrochloric acid 0.1 N to pH 2.5. In another beaker, weigh the sulfonyl β-cyclodextrin, add half of the amount of purified water, and stir until well mixed. Add the cyclodextrin dispersion to the itraconazole mixture under constant stirring. Add the hydroxypropyl cellulose slowly and stir for 30 min. Let it hydrate for 24 h. Fill it purified water until the final volume and stir slowly for 30 min. A viscous white suspension was obtained [24,25,26,27,28].
- Formulation E: Weigh the required amount of itraconazole and combine it with polysorbate 80. Add the polyethylene glycol 400 and mix well. In another beaker, weigh the sulfonyl β-cyclodextrin, add half of the amount of purified water, and stir until well mixed. Add the cyclodextrin dispersion to the itraconazole mixture under constant stirring. Add the hydroxypropyl cellulose slowly and stir for 30 min. Let it hydrate for 24 h. Fill it with purified water until the final volume and stir slowly for 30 min. Adjust the pH of the mixture with hydrochloric acid 0.1 N to pH 1.9. A viscous white suspension was obtained [24,25,26,27,28,29].
- Formulation F: Weigh the required amount of itraconazole and combine it with polysorbate 80. Add the polyethylene glycol 400 and mix well. In another beaker, weigh the amounts of sulfonyl β-cyclodextrin and sodium benzoate required, mix with half of the amount of purified water required, and stir until well mixed. Add the cyclodextrin dispersion to the itraconazole mixture under constant stirring. Add the hydroxypropyl cellulose slowly and stir for 30 min. Let it hydrate for 24 h. Fill it with purified water until the required volume and stir slowly for 30 min. Adjust the mixture pH with hydrochloric acid 0.1 N to pH 1.9. A viscous white suspension was obtained [24,25,26,27,28,29].

Three batches of each formulation were prepared. An aliquot of each one was taken, and they were characterized in terms of osmolarity and final pH. Then, formulations were kept in the fridge under controlled temperature (2–8 °C) until evaluation according to the following assays.

### 2.3. Organoleptic Characteristics

For all the formulations, it was observed if there was any alteration in their organoleptic characteristics during the time of the assay. Characteristics like odor and color were evaluated.

### 2.4. Physicochemical Characteristics

The osmolarity of the aliquots was measured with the osmometer Osmomat^®^ 030 Gonotec GmbH (Advanced Instruments SAS, distributed by Proquilab, Murcia, Spain). The pH of the samples was measured with the pH meter Fisher Scientific Accumet AE150 (Fisher Scientific S.L., Madrid, Spain) following the method 2.2.3 of Real Farmacopea Española (RFE) 2015 [30].

### 2.5. Stability

Physical and chemical stabilities of all the formulations, A, C, D, and F, were evaluated for 30 days on days 0, 1, 4, 7, 11, 15, 20, and 30.

At the sampling time, the sediment was observed, and its redispersibility was verified. Then, 0.750 mL of each suspension was taken after mixing the samples properly and was vigorously mixed with 0.750 mL of ACN. Afterwards, samples were centrifuged and diluted with ACN (20:980, sample: ACN), and the amount of itraconazole was measured by (high-performance liquid chromatography) HPLC. HPLC analysis was performed using a UV detector (Waters^®^ 2487, Waters Cromatografia S.A. Cerdanyola del Vallès, Spain) at 262 nm, an X-Bridge^®^ (Waters Cromatografia S.A. Cerdanyola del Vallès, Spain) C18 column (3.5 μm, 4.6 × 100 mm), a mobile phase of 40% methanol, 15% acid water (0.05% *v*/*v* TFA in water), and 45% acetonitrile with a flow rate of 1 mL/min and a temperature of 30 °C [30,31,32,33,34]. All analytical methods were validated and demonstrated to be adequate regarding linearity, accuracy, precision, selectivity, and specificity.

The shelf life was characterized by the lower limit of the 90% confidence interval of T_90_.

### 2.6. Dissolution Assays

The dissolution profiles at pHs 1.2, 4.5, and 6.8 of itraconazole from the reference product and formulations A, B, C, and D were obtained using a paddle apparatus (PT-DT70 Pharma Test^®^ Pharma Test Apparatebau AG, distributed by Proquilab, Murcia, Spain) in 300 mL of solution at 50 rpm and 37 °C. Buffer solutions were prepared as follows: pH 1.2 contained sodium chloride 50 mM and hydrochloric acid 0.1 N; pH 4.5 was made with sodium acetate 36.5 mM; and pH 6.8 contained dipotassium phosphate 50 mM. Samples were taken at 5, 10, 15, 20, 30, 40, 45, 60, 90, and 120 min, and after being centrifuged, the supernatant (diluted 1:1 with the same buffer solution) was analyzed by HPLC, as mentioned above [30,31,32,33,34].

The dissolution profiles with pre-digestion (dumping test) were obtained for the reference product and formulations D, E, and F. In this case, 2 mL of the suspensions was first added to 50 mL of buffer solution at pH 1.2 (sodium chloride 50 mM, hydrochloric acid 0.1 N), and samples were taken at 5, 10, 15, and 20 min; then, after measuring the pH, 52 mL was poured down into 450 mL of buffer solution at pH 6.8 (dipotassium phosphate 50 mM). After readjusting the pH of the mixture to pH 6.8, samples were taken, one immediately and others at 35, 40, 45, 60, 90, and 120 min. All samples were centrifuged and diluted 1:1 with the buffer before to be analyzed by HPLC [35].

### 2.7. Permeability

The permeability of itraconazole from the reference product and tested formulations was evaluated in rats using an in situ closed-loop perfusion technique (adapted from the Doluisio technique) [36,37].

First, a surgery was performed on the animal, and a loop of the small intestine was isolated. Then, 2 mL of each suspension (10 mg/mL) was mixed with 8 mL of buffer solution at pH 6.8 (dipotassium phosphate 50 mM), and they were introduced into the isolated loop. At 5, 10, 15, 20, 25, and 30 min, 200 µL samples were taken, and after being centrifuged, they were analyzed by HPLC.

The luminal concentrations of itraconazole at each time (C) obtained after the HPLC analysis of samples were used to obtain the apparent absorption rate constant (k_app_) and the apparent intestinal permeability (P_app_) with equations 1 and 2, in which C_0_ is the extrapolated concentration of itraconazole at time 0, and R is the radius of the intestine [38]. Statistical differences were evaluated by means of a one-way analysis of variance test (ANOVA), followed by a Scheffe pot hoc test. A statistical significance level of 0.05 was used. Tests were performed using SPSS (V 26.0).

(1) $$C=C_{0}\cdot e^{-k_{app}\cdot t}$$

(2) $$P_{app}=k_{app}\cdot \frac{R}{2}$$

The permeability studies were approved by the Scientific Committee of the Faculty of Pharmacy, Miguel Hernandez University and followed the guidelines described in the EC (European Community) Directive 86/609, the Council of the Europe Convention ETS 123 (European Convention for the Protection of Vertebrate Animals Used for Experimental and Other Scientific Purposes), and Spanish national laws governing the use of animals in research (A1330354541263).

## 3. Results

### 3.1. Organoleptic Characteristics

All the elaborated formulations were odorless and had a homogeneous white color. They were viscous in texture. No particles in the suspension were visually observed, except in formulation A.

Since no flavoring agents were added, no aroma was expected. Nevertheless, the compounds that contained polysorbate 80 had its characteristic smell.

### 3.2. Physicochemical Characteristics

Table 2 summarizes the osmolarity and the final pH of each formulation. It can be seen that the reference product was an acidic formulation (pH = 1.88).

Formulation A, the only one not buffered, had the highest pH (6.02), while the pH of formulation B was due to the buffer capacity of Syrpend^®^, the base in which itraconazole was dispersed. In formulations C and D, the pH was adjusted to 2.5 when itraconazole was mixed with propylene glycol (C) or PEG 400 and polysorbate 80 (D), respectively, before adding the cyclodextrin dispersion. As can be seen in Table 2, the final pHs of these two formulations were higher, around 3.7. For this reason, the pH was adjusted in formulations E and F and at the end of the elaboration process to emulate the reference formulation pH.

The reference product was clearly hyperosmolar, compared to blood, as were formulations A and C. Formulations D, E, and F had about 500 mOsm/kg, while formulation B had a very low content in osmotically active substances.

### 3.3. Stability

**Figure 1:**
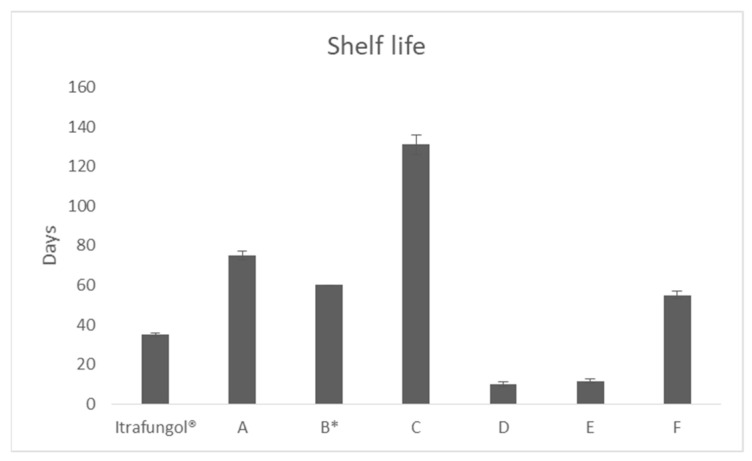
Shelf life of the different formulations of itraconazole tested. B*: Theoretical data obtained from the available stability studies and the compatibility tables offered by the manufacturer about itraconazole 20 mg/mL in Syrspend pH 4.

According to the information provided by the manufacturer of the reference product, it should not be used for longer than 5 weeks, meaning 35 days. Figure 1 shows the results of the stability test of the formulations prepared, which were calculated using T_90_. As can be seen, compounds B, D, and E were less stable than the reference product, while formulas A, C, and F were stable for more than 50 days.

### 3.4. Dissolution Studies

**Figure 2:**
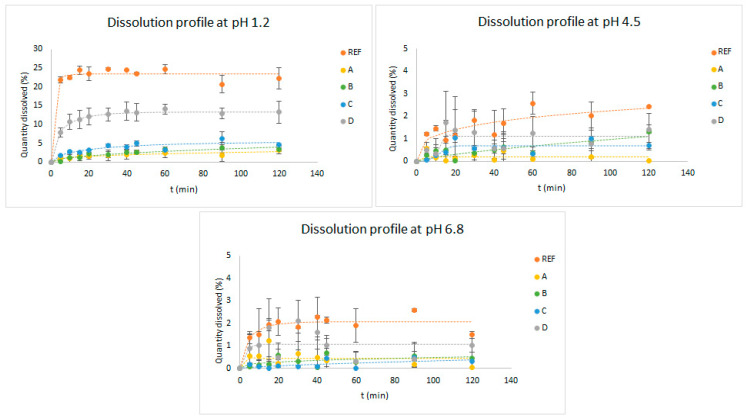
Dissolution profiles of itraconazole from the reference product and from formulations A, B, C, and D at pHs 1.2, 4.5, and 6.8. Results are as a percentage of the dose.

Figure 2 shows the profiles of the dissolution tests of the formulations assayed (Reference, A, B, C, and D), carried out with the paddles test at pHs 1.2, 4.5, and 6.8.

As shown in Figure 2, the dissolution profiles of formulations A, B, and C were different with respect to the reference. From formulations A–C, the maximal percentage of the dose dissolved during the assay was below 1% at all pHs, while from the reference product, 2% of the dose dissolved at pHs 4.5 and 6.8; nearly 25% dissolved at pH 1.2. Formulation D had a higher dissolved amount than A–C, but it was not enough to be similar to the reference product. In formulation D, 13% of the dose dissolved at pH 1.2, and over 1% dissolved at pHs 4.5 and 6.8.

Due to the similarity of the dissolution profile of formulation D with the reference product, some adjustments were made to prepare two other formulations (E and F). These later formulations were assayed through the dumping test.

**Figure 3:**
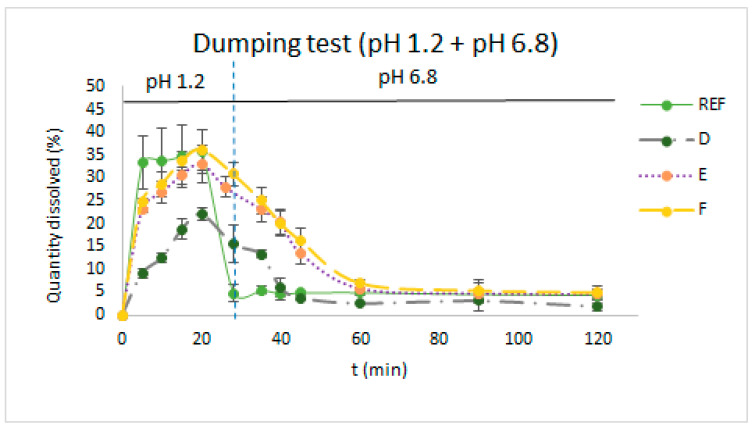
Dissolution profiles of itraconazole from the reference product and formulations D, E, and F obtained using the dumping test technique. Results are as a percentage of the dose.

In Figure 3, dissolution profiles of itraconazole from the reference product and formulations D, E, and F were obtained using the dumping test technique. This technique is the closest to what happens in the digestive tract.

Results are as a percentage of the dose. In Figure 3, dissolution profiles of formulas D, E, and F were obtained using the dumping test technique. Although formulations D, E, and F had dissolution percentages in the gastric environment (pH 1.2) of around 30 and 35 percent of the prepared dosage, and although there was no precipitation at pH 6.8, the amount available for absorption was less than the theoretical dosage.

### 3.5. Permeability Assay

**Figure 4:**
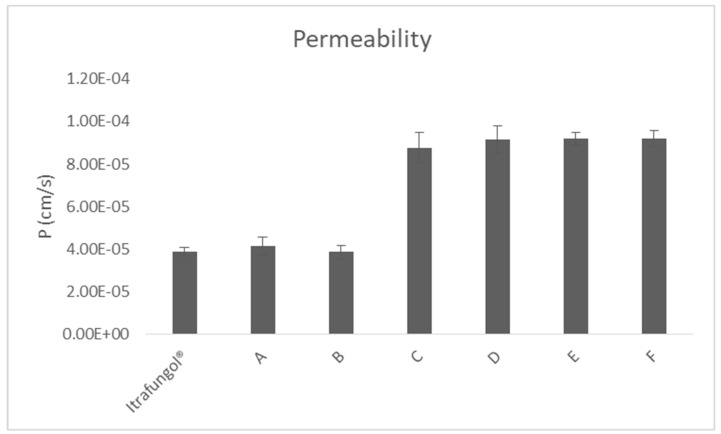
Permeability of itraconazole (P) through the small intestine of rats, from the reference product and from the formulations prepared.

Permeability values of itraconazole from the different formulations are summarized in Figure 4.

In Figure 4, it can be observed that itraconazol permeability from the reference product was 4 × 10^−5^ cm/s, similar to that obtained from formulations A and B. In compounds C to F, which were prepared with β-cyclodextrin, higher permeabilities were observed that were statistically different from the reference product, reaching double values, compared with those obtained with Itrafungol^®^.

## 4. Discussion

Itraconazol is a weak base with a pKa of 3.7 and a solubility dependent upon the pH. (In 0.1 M chlorhydric acid (HCl), it is 4–6 µg/mL, and in water, it is 1–4 ng/mL.) It is also a lipophilic compound, with a log P (n-octanol/water) of 5.66 at pH 8.1 [39,40]. The aspects mentioned are important when preparing oral formulations. A suspension was selected instead of a solid dosage form because it was considered a better option in veterinary medicine, since it facilitates administration.

In the reference product, propylene glycol (E-1520) was used as humectant, sorbitol 70% and sodium saccharine were used as sweeteners, hydroxypropyl β-cyclodextrin was used as a solubilizer, and hydrochloric acid and sodium hydroxide were used for pH adjustment. In its composition, cherry flavoring and purified water as a vehicle were also included. Taking this as a guide, the first approaches, formulations A–D, were assayed and then refined to prepare formulations E and F. However, although flavoring agents can improve palatability [16,41], none of the compounds made included them. When using flavoring agents, it is important to check their suitability for the species of destiny because some of them include products like ethanol or other substances to dissolve them, which can be dangerous for the animals. In this sense, there are no flavorings suitable for cats in the Spanish market [16,42].

In formulation A, propylene glycol was employed as a humectant, and sodium carboxymethylcellulose was employed as a suspending agent. Sorbitol 70% non-crystallizable was selected as a sweetener. It should be noted that the suitable sweeteners for veterinary medicine are saccharin or sorbitol, since, for example, xylitol can cause severe hypoglycemia in dogs, due to the massive generation of insulin by the pancreas [43]. The pH was not adjusted.

Formulation B was prepared with a pre-prepared excipient (Syrspend Dry pH4^®^) considered suitable in veterinary practice. This excipient is a patented formulation which does not contain preservatives or flavoring agents but contains sucralose as a sweetener and thickening agents. Syrspend Dry pH 4 ^®^ is indicated to prepare formulations for actives that require an acidic pH of around pH 4–5. Each unit contains 13 g of powder excipients, and it is served into a graduated dispensing 200 mL amber PET bottle [17].

To prepare formulation C, sulfonyl β-cyclodextrin was selected [24,25,26]. This kind of cyclodextrin improves itraconazole solubility and masks itraconazole flavor. This preparation was made following protocol 2568 of Compounding Today [44]. In this case, the pH was set to 2.5 in the mixture of itraconazole and propylene glycol, with an HCl of 0.1 M to dissolve the active ingredient. The final pH of the suspension was not adjusted.

When preparing formulation D, the protocol used was the same as for formulation C, but polyethylene glycol 400 was used as the solubilizer and suspensor agent. Polysorbate 80 was used as the humectant, and the pH was fixed with an HCl 0.1 M solution. Hydroxypropyl methylcellulose was used as the gelling agent because it is stable at the final pH [24,25,26,27,44].

Formulation E has the same composition as D, but in this preparation, the pH was adjusted to 1.9 once prepared to emulate the pH of the reference product [24,25,26,27,29,44].

Finally, formulation F differed from E in the inclusion of a preservative agent to increase the shelf life of the product. The preservative selected was sodium benzoate because it is stable at a low pH, and it can be used in veterinary products. The amount of sodium benzoate was limited to avoid the final product being hyperosmolar [24,25,26,27,29,44].

All formulations were bottled in amber glass bottles of 60 mL (multidose container), except formulation B, which was bottled in its original container.

Once prepared, it was observed that the reference formulation and formulations A and C had a very high osmolar concentration. That can be attributed to the sugars or polyols in their composition, typically used as sweeteners and co-solvers. From that perspective, since this situation can generate intestinal disorders, it was considered a better option to eliminate those kinds of compounds on formulations B, D, E, and F and choose other ingredients for better compatibility [15,16,27,41].

Suspensions are unstable by nature, even when gelling agents are added. Due to the particle size and the poor solubility of the active, the system tends to flocculate [23]. To maintain the suspensions as it was initially and reduce as much sedimentation as possible, gelling agents were added to give stability. Nevertheless, two of the formulations assayed, B and C, did not include gelling agents.

When freshly prepared, all were viscous white suspensions. After a while and depending on the composition, sedimentation occurred in all formulations at different rates; however, all sediments redispersed immediately by shaking. The dose quantified at any time after shaking was homogeneous and consistent with the theoretical concentration of the active in the formulations.

Regarding pH, general protocols for suspensions do not include checking the final pH as a control [22]. Since there are no studies for this parameter in veterinary compounding, the available literature for liquid forms of oral administration were used. These publications indicate that the measurement of pH should be established as mandatory, and it should be included in the modus operandi of the production protocols [22,41]. Although in formulations A–D, the pH was not controlled, the pH depends on the excipients used in formulations to dissolve itraconazole, as established in the protocol used [44]. However, it was observed that the resulting pH of the formulations rose to approximately 3.7. From the dissolution tests at the three pH values (Figure 2), it was observed that the dissolution profile of formulation D was the closest to that of the reference formulation; nevertheless, the amount of itraconazole dissolved from formulation D at the end of the assays was lower. For this reason, in formulations E and F, it was decided to adjust the pH to about 1.9 at the end of the elaboration process.

As mentioned, itraconazole is a BCS (Biopharmaceutics Classification System) class II drug, has low solubility, is pH-dependent, and has high permeability, so a critical point when administered orally is the change in pH when the contents of the stomach are transferred into the intestinal tract. For this reason, one of the most adequate in vitro dissolution tests to predict in vivo behavior is the dumping test. This test is the combination of pre-digestion in acid media and the simulation of gastric empty in duodenum with the change in pH. As can be seen in Figure 3, formulations D, E, and F showed a slower precipitation rate than the reference formulation in the dumping dissolution test when changing pH from 1.2 to 6.8. Formulations E and F and the reference product, those that had the same final pH, were the ones able to provide the highest percentage dissolved. According to the results observed, when using this dissolution medium, formulation F seemed to be the most promising one. The combination of excipients used enabled the same amount of itraconazole to be dissolved at pH 1.2 as the reference product, but in contrast to the reference formulation, the precipitation of itraconazole upon leaving the stomach was delayed. The latter can be attributed to the presence of HPMC, which increases the viscosity of the formulation [26,45].

Itraconazole has poor solubility in water. To overcome this inconvenience, the addition of a cyclodextrin into the formulation was considered worthy. In the compounding protocols, 2-hydroxypropyl β-cyclodextrin is recommended; nevertheless, since itraconazole solubility is higher in acidic medium, and in this condition, sulfonyl β-cyclodextrin is more stable, this was considered a better option [27,46,47,48,49]. Cyclodextrins are a group of cyclic oligosaccharides that contain six or more glucopyranose monomers linked via α-1,4-glycosidic bonds. CDs, through their slightly apolar internal cavities, act as complexation agents for a broad range of lipophilic molecules. This way, CDs allow the increasing aqueous solubility of lipophilic drugs. Due to that characteristic and others like increased stability of the complex or increased bioavailability of the drug included, CDs are widely used in compounding and in the food industry. [46,47] 

The results of the permeability studies showed that itraconazole permeability was increased more than two-fold, due to β-cyclodextrin presence (formulations C, D, E, and F), with respect to itraconazole from the reference and the other formulations without cyclodextrins in their composition (A and B). The main difference between the reference product and formulations C–F was the cyclodextrin used. The amount of cyclodextrin in the reference product is unknown. In the formulations we prepared, the cyclodextrin was added at 25%, as described for 2-hydroxypropyl beta cyclodextrin in compounding protocols [44].

In summary, according to the results obtained, the aspects that were considered more relevant were the final pH (around 2) and the presence of cyclodextrins.

Depending on the excipients used and the elaboration protocols, the stability of some compounded itraconazole suspensions can differ from 14 to 60 days [17,44,50]. Even the stability test showed that formulation C was the one with higher stability (131 days ±5 days), due to the results of solubility and permeability assays; it can be considered that formulation F could be the most appropriate. Formulation E, although similar to formulation F in the dissolution and permeability assays, was less stable. Taking into account that this formulation was prepared manually, its stability can be considered satisfactory (55 ± 2 days when stored between 2–8 °C), as it was slightly more stable than the reference formulation.

Formula F is an improvement of Formula C, which, in the original protocol, can be used in veterinary practice [44]. Recently, it has been published that polysorbate 80 produces cardiovascular changes and hypersensitivity effects in dogs when given orally at 10 mg/kg, while it is safe for cats at punctual administrations [51,52]. This latter fact points out the relevance of the composition of a formulation for veterinary healthcare. In spite of the good results obtained with Formula F, its use has to be restricted to some species, such as cats. Due to the importance of and differences in the effects of the components in different species, this formula has to be evaluated ingredient by ingredient to assure security for other target species.

## 5. Conclusions

A new formulation for itraconazole oral administration was obtained using a combination of excipients, sulfonyl β-cyclodextrin, and HPMC. This preparation dissolves amounts as high as the reference product Itrafungol^®^ at pH 1.2 and delays the precipitation of itraconazole upon leaving the stomach. The most promising formulation contained hydroxypropyl methylcellulose and β-cyclodextrin. This combination of excipients can dissolve the same concentration as the reference product and delays the precipitation of itraconazole upon leaving the stomach. Moreover, intestinal permeability of itraconazole was increased more than two-fold.

Due to the increased permeability that occurs, compared to the reference product, the possibility of an increased risk of toxicity should be evaluated. To avoid this risk, a dosage adjustment or a longer dosing interval can be considered to overcome this enhanced absorption. Reducing the concentration of cyclodextrin could also be considered to reduce the absorption of itraconazole.

## Figures and Tables

**Table 1:** Composition of the reference product (Itrafungol^®^) and the different itraconazole compounds. qs: quantum satis.

Product	REF	A	B	C	D	E	F
Itraconazole	1%	1%	1%	1%	1%	1%	1%
Caramel E150	0.02%						
Cherry flavor	✓						
HCl 37%	✓						
HCl 0.1 M				qs	qs	qs	qs
Hydroxypropyl β-cyclodextrin	✓						
Sulfonyl β-cyclodextrin				25%	25%	25%	25%
HPMC					1.5%	1.5%	1.5%
PEG 400					10%	10%	10%
Polysorbate 80					5%	5%	5%
Propylene Glycol	10.36%	10%		10%			
Sodium benzoate							0.025%
Sodium carmellose		0.5%					
Sodium hydroxide	✓						
Sodium saccharin	✓						
Sorbitol 70%	24.5%	25%					
Syrspend pH 4 Dry			6.5%				
Purified water	✓	qs	qs	qs	qs	qs	qs

qs to dissolve itraconazole before adding the cyclodextrin solution. ✓ the reference formulation contains this compound.

**Table 2:** pH and osmolarity of the different compounds of itraconazole.

	REF	A	B	C	D	E	F
Final pH	1.88 ± 0.01	6.02 ± 0.01	4.41 ± 0.01	3.70 ± 0.01	3.62 ± 0.01	1.90 ± 0.01	1.98 ± 0.01
Osmolarity (mOsm/kg)	1824 ± 2	1107 ± 1	63 ± 1	1608 ± 2	547 ± 1	493 ± 1	499 ± 1

## Data Availability

Ruiz-Picazo, Alejandro; Bermejo, Marival; González-Álvarez, Isabel; González-Álvarez, Marta; Sánchez-Dengra, Bárbara (2024), “Itraconazole Data (UMH)”, Mendeley Data, V2, https://data.mendeley.com/datasets/nbx8s2jt6w/1.
